# Primary Posterior Tracheopexy in Esophageal Atresia Decreases Respiratory Tract Infections

**DOI:** 10.3389/fped.2021.720618

**Published:** 2021-09-09

**Authors:** E. Sofie van Tuyll van Serooskerken, Stefaan H. A. J. Tytgat, Johannes W. Verweij, Arnold J. N. Bittermann, Saskia Coenraad, Hubertus G. M. Arets, David C. van der Zee, Maud Y. A. Lindeboom

**Affiliations:** ^1^Department of Pediatric Surgery, Pediatric Wilhelmina Children's Hospital, University Medical Center Utrecht, Utrecht, Netherlands; ^2^Congenital Esophageal and Airway Team Utrecht, Pediatric Wilhelmina Children's Hospital, University Medical Center Utrecht, Utrecht, Netherlands; ^3^Department of Pediatric Otorhinolaryngology, Pediatric Wilhelmina Children's Hospital, University Medical Center Utrecht, Utrecht, Netherlands; ^4^Department of Pediatric Pulmonology, Pediatric Wilhelmina Children's Hospital, University Medical Center Utrecht, Utrecht, Netherlands

**Keywords:** esophageal atresia, tracheomalacia, bronchoscopy, thoracoscopy, posterior tracheopexy, brief resolved unexplained event, respiratory tract infection

## Abstract

**Background:** Esophageal atresia (EA) is often accompanied by tracheomalacia (TM). TM can lead to severe respiratory complaints requiring invasive treatment. This study aims to evaluate if thoracoscopic primary posterior tracheopexy (PPT) can prevent the potential sequelae of TM in patients with EA.

**Methods:** A cohort study including all consecutive EA patients treated between 2014 and July 2019 at the Wilhelmina Children's Hospital was conducted. Two groups were distinguished: (group 1) all EA patients born between January 2014 and December 2016 and (group 2) all EA patients born between January 2017 and July 2019, after introduction of PPT. In the latter group, PPT was performed in EA patients with moderate (33–66%) or severe (67–100%) tracheomalacia, seen during preoperative bronchoscopy. Group differences were assessed using the Fisher's exact test for bivariate variables and the Mann–Whitney *U*-test for continuous variables.

**Results:** A total of 64 patients were included in this study (28 patients in group 1; 36 patients in group 2). In group 2, PPT was performed in 14 patients. Respiratory tract infections (RTIs) requiring antibiotics within the first year of life occurred significantly less in group 2 (61 vs. 25%, *p* = 0.004). Brief resolved unexplained events (BRUEs) seemed to diminish in group 2 compared to group 1 (39 vs. 19%, *p* = 0.09).

**Conclusion:** Thoracoscopic primary posterior tracheopexy decreases the number of respiratory tract infections in EA patients. The clinical impact of reducing RTIs combined with the minimal additional operating time and safety of PPT outweighs the risk of overtreatment.

## Introduction

In up to 87% of patients, esophageal atresia (EA) can be associated with some form of tracheomalacia (TM) (1). TM can be caused by flaccidity of the cartilaginous anterior rings, a floppy posterior membrane, or both and may lead to a dynamic collapse of the tracheal lumen (2, 3). This collapse of the trachea can result in a wide spectrum of symptoms and sequelae ranging from mild complaints, such as stridor or wheezing, to brief resolved unexplained events (BRUEs) (3). Furthermore, collapse of the trachea may lead to an ineffective cough and impaired clearance of secretions, increasing the risk of respiratory tract infections (4, 5). In severe TM, invasive treatment might be warranted (6, 7). Surgical treatment of preference depends on the type of TM and includes aortopexy to lift the aortic compression on the anterior flaccid cartilaginous rings (3, 8), or posterior tracheopexy of the floppy membrane to prevent posterior tracheal intrusion (9). In a previous study, we have introduced a new approach in which a posterior tracheopexy is performed during the thoracoscopic correction of EA. Results showed this approach to be feasible (10).

The aim of this study is to evaluate if thoracoscopic primary posterior tracheopexy (PPT) can prevent the potential respiratory sequelae of tracheomalacia in patients with EA and concurrent TM.

## Methods

### Study Design and Participants

A cohort study including all consecutive EA patients between January 2014 and July 2019 was conducted at the University Medical Center Utrecht, Wilhelmina Children's Hospital. The variables of interest were collected prospectively at standardized time points for all children according to standard clinical practice. The comparative design was applied after data collection. Patients were excluded for follow-up if they had died before the age of 1 year. In January 2017 thoracoscopic PPT for moderate or severe TM was introduced in our hospital. Two patient subgroups were distinguished: (group 1) all EA patients born between January 2014 and December 2016 and (group 2) all EA patients born between January 2017 and July 2019, after the introduction of PPT. Data of patients that underwent thoracoscopic PPT were prospectively collected.

### Surgical Procedure

**Figure 1:**
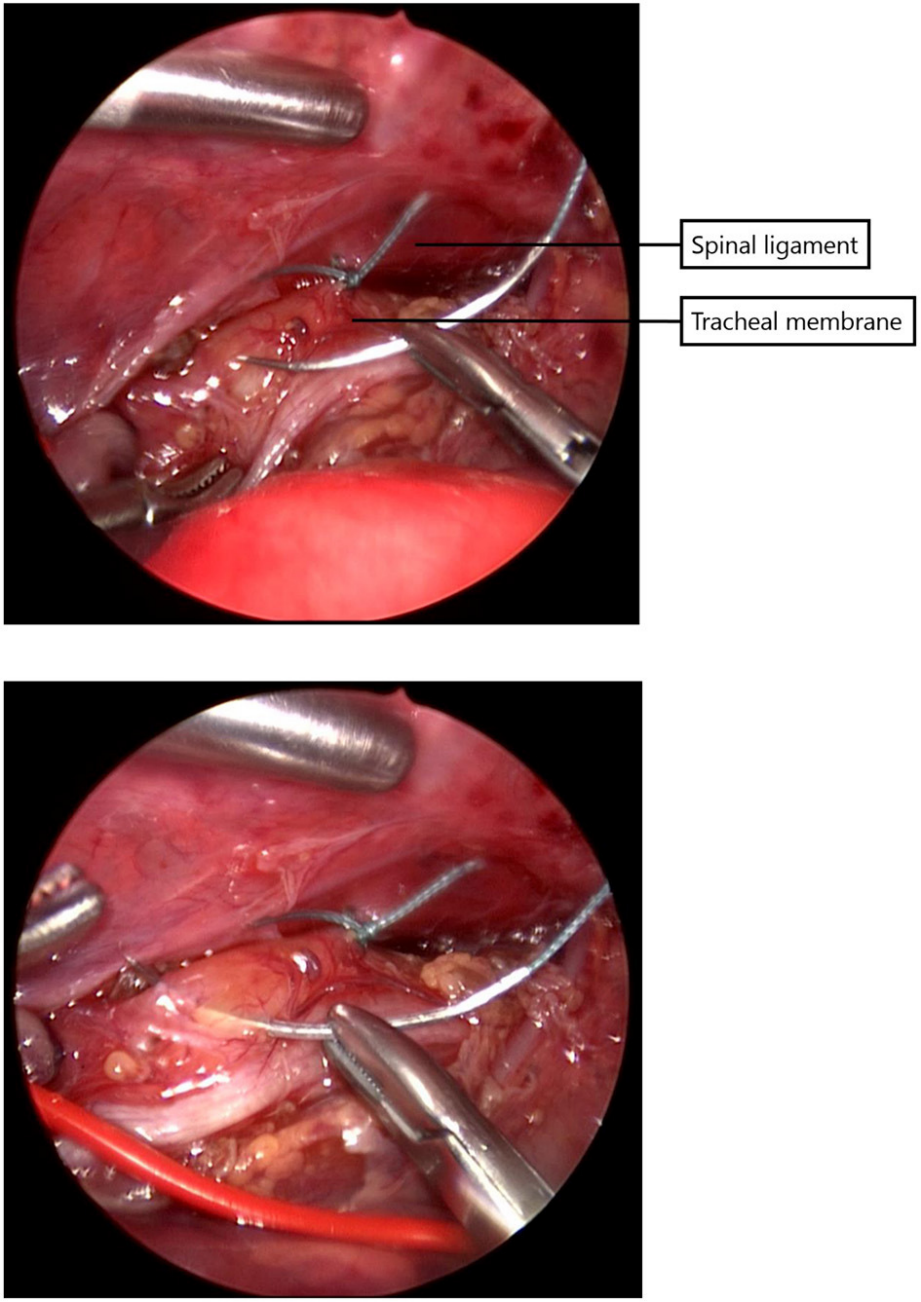
The posterior tracheal membrane is fixed to the the anterior spinal ligament with one to three non-absorbable sutures.

**Figure 2:**
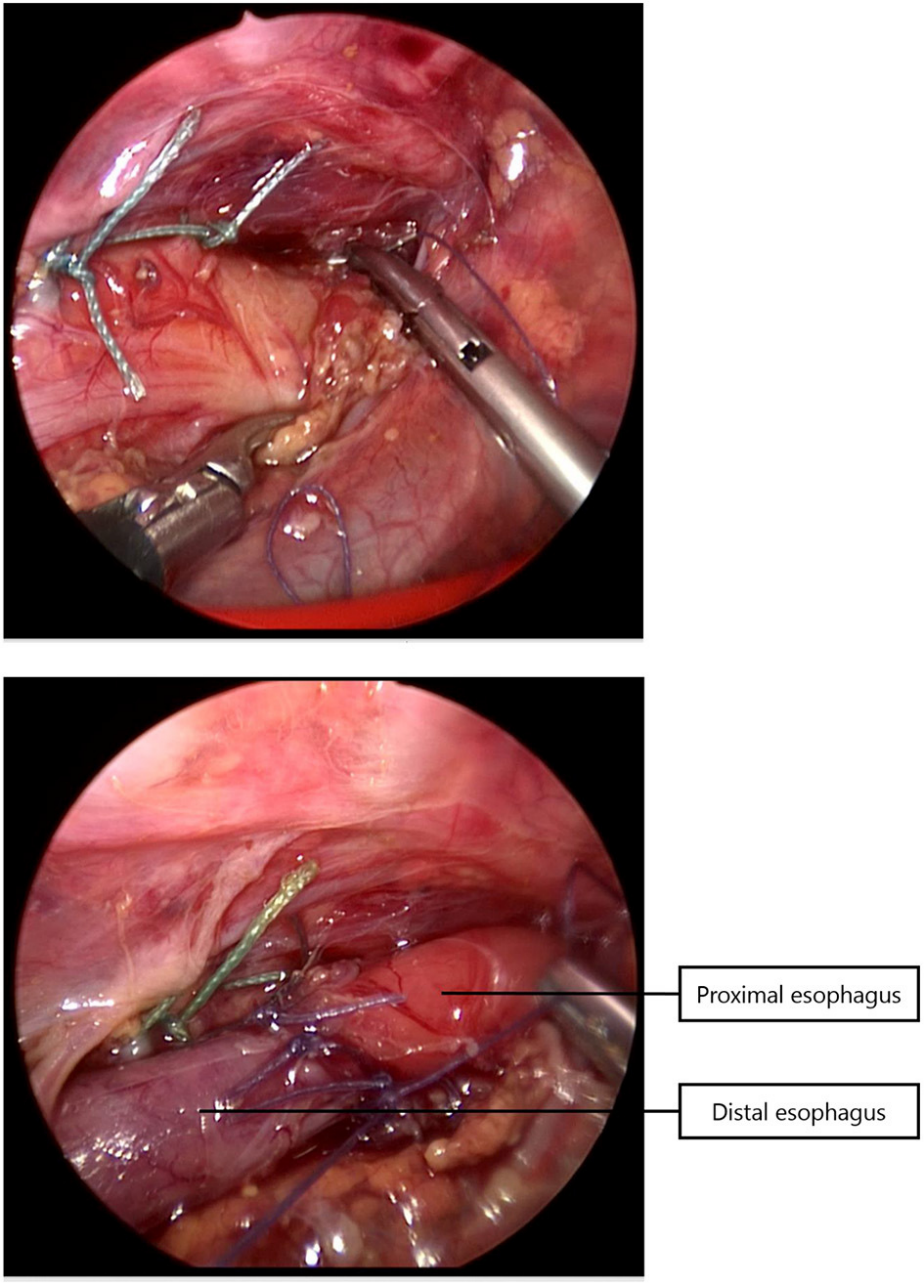
After performing the tracheopexy, the proximal and distal esophagus are anastomosed.

Since 2014 almost all infants with EA underwent a rigid bronchoscopy prior to surgery to evaluate the presence of TM and to exclude a proximal fistula. Since 2017, a standardized scoring system for TM has been introduced (11). Patients with moderate to severe tracheomalacia were eligible for PPT. Tracheal obstruction, evaluated by bronchoscopy, was defined as collapse of the tracheal wall at three different levels, the upper third, middle third, and lower third of the trachea (11). TM was considered moderate when the tracheal lumen collapsed 33–66% and severe when 67–100%. The surgical procedure of thoracoscopic PPT during esophageal repair has been described previously (10). In short, during the procedure for thoracoscopic esophageal repair, the posterior tracheal membrane is fixed to the anterior spinal ligament with one to three non-absorbable sutures (Figure 1), prior to the anastomosis of the esophageal ends (Figure 2).

### Clinical Assessment

All baseline characteristics, including gender, type of EA and associated anomalies, and all surgical data including age at time of surgery, postoperative complications and length of hospital and NICU stay, were collected. Prospective data of patients that underwent PPT was obtained during standard EA follow-up in outpatient care at 4 weeks and 3, 6, 12 months of age.

#### Respiratory Outcome

Patients underwent standardized clinical assessment regarding respiratory symptoms. The primary outcome measures were respiratory symptoms, including respiratory tract infections (RTIs) requiring antibiotics within the first year of life and occurrence of BRUEs. Diagnosis of the RTIs was made by the pediatric pulmonologist. The pediatric pulmonologists based their decision on symptpoms and/or chest X-rays. Antibiotic treatment was prescribed by the pediatric pulmonologist. BRUE is defined as an event in which an infant younger than 1 year old presents with cyanosis, pallor, altered breathing, hypotonia or hypertonia and/or altered responsiveness (12).

### Statistical Analysis

Continuous variables were presented as median and range and categorical data were presented as frequencies and percentage. To assess group differences for bivariate variables the Fisher's exact test was used. Group differences for continuous variables were assessed using the Mann-Whitney *U*-test. A *p* < 0.05 was considered significant. The analyses were performed with SPSS for Windows, version 25.0 (IBM Corp., Armonk, NY).

### Ethical Approval

This cohort study was submitted to the UMCU Ethics Committee. No ethical approval was required according to the Medical Research Involving Human Subject Act. The study was carried out in accordance with the Declaration of Helsinki.

## Results

In total, 67 consecutive EA patients were admitted at the Wilhelmina Children's Hospital between January 2014 and July 2019. Three patients that died within the first weeks after birth were excluded from further analysis. One patient died before esophageal repair due to severe prematurity with pulmonary bleeding. Two patients died after esophageal repair due to causes unrelated to surgery (cardiac anomalies and cerebral abscesses). The 64 remaining patients were all evaluated in our outpatient clinic at 4 weeks and 3, 6, 12 months. The patients were divided into two groups: the first group, before the introduction of PPT, consisted of 28 consecutive patients admitted between 2014 and 2016 (group 1). The second group, after the introduction of PPT, consisted of 36 patients admitted between 2017 and 2019 (group 2). Patient characteristics were comparable between the two groups, as shown in Table 1.

**Table 1:** Patient characteristics of group 1 and group 2.

**Variable**	**Group 1** **(2014–2016)**	**Group 2** **(2017–2019)**	***p*-value**
	***n* = 28**	***n* = 36**	
Male	16 (57%)	24 (67%)	0.45
Gestational age (weeks)	39.2 (31.6–41.6)	37.3 (28.3–42.3)	0.08
Premature	7 (25%)	16 (44%)	0.12
Twin	3 (11%)	2 (6%)	0.65
Birthweight (g)	2,755 (1,585–4,170)	2,692 (1,050–3,950)	0.37
**Apgar score**			
1 min	8 (2–9)	9 (3–10)	0.17
5 min	9 (3–10)	9 (5–10)	0.48
**Type EA**			
A	3 (11%)	1 (3%)	0.38
B	1 (3.6%)	0	
C	23 (82%)	32 (89%)	
D	0	2 (5.6%)	
E	1 (3.6%)	1 (2.8%)	
**Associated anomalies^#^**			
Trisomy 21	2 (7%)	1 (3%)	0.57
VACTERL	6 (21%)	8 (22%)	1.0
Musculoskeletal	10 (36%)	15 (42%)	0.80
Urogenital	8 (29%)	5 (14%)	0.33
Cardiovascular	12 (43%)	15 (42%)	1.0
Gastrointestinal	4 (14%)	2 (6%)	0.40

*EA, esophageal atresia*.

*All data are presented as median (range) or n (%)*.

# *Some patients had multiple anomalies*.

In group 2, a PPT was performed in 14 patients (39%). Of these 14 patients, 12 patients (86%) had EA Gross type C, and 2 patients (14%) type D. Patient characteristics are presented in Table 2. There were no relevant significant differences between the no-PPT patients and the PPT patients within group 2.

**Table 2:** Patient characteristics of group 2 (2017–2019).

**Variable**	**No-PPT,** ***n* = 22**	**PPT,** ***n* = 14**	***p*-value**
Male	14 (64%)	10 (71%)	0.73
Gestational age (weeks)	36.7 (29.1–42.3)	38.4 (28.3–41.4)	0.35
Premature	12 (55%)	4 (29%)	0.18
Twin	2 (9%)	0 (0%)	0.51
Birthweight (g)	2,249 (1,220–3,950)	3,008 (1,050–3,550)	0.22
**Apgar score**			
1 min	9 (4–10)	8.5 (3–9)	0.84
5 min	9 (7–10)	9 (5–10)	0.74
**Type EA**			
A	1 (4.5%)	0	0.20
C	20 (91%)	12 (86%)	
D	0	2 (14%)	
E	1 (4.5%)	0	
**Associated anomalies^#^**			
Trisomy 21	1 (4.5%)	0	1.0
VACTERL	3 (14%)	5 (36%)	0.22
Musculoskeletal	10 (45.5%)	5 (36%)	0.73
Urogenital	0	5 (36%)	0.005^*^
Cardiovascular	9 (41%)	6 (43%)	1.0
Gastrointestinal	1 (4.5%)	1 (7%)	1.0

*PPT, primary posterior tracheopexy; EA, esophageal atresia*.

*All data are presented as median (range) or n (%)*.

# *Some patients had multiple anomalies*.

* *Indicating statistical significance*.

### Surgical Outcome

Overall analyses of group 1 and group 2 showed no significant differences in age at EA surgery, postoperative NICU time, length of hospital stay or anastomotic leakage between the two groups (Table 3A). In group 2, moderate to severe tracheal collapse was diagnosed in 13 patients (Table 4). In two patients with mild TM (20% tracheal collapse) on bronchoscopy, increased flaccidity of the posterior tracheal membrane was seen during thoracoscopy after ligation and transection of the distal tracheoesophageal fistula. Therefore, a PPT was also performed in these two patients. The middle and distal third of the trachea were most often affected with a median tracheal collapse of 50% (range 20–90%). Thoracoscopic PPT was uncomplicated and successful in all patients with a median of 2 sutures (range 1–3). Median time per suture was 6 min (range 4–12 min). Anastomotic leakage occurred in 21% and could be treated conservatively in all patients. There were no significant differences in surgical outcome between patients with or without PPT in group 2 (Table 3B).

**Table 3A:** Surgical data of group 1 and group 2.

**Variable** **(median, range)**	**Group 1** **(2014–2016)**	**Group 2** **(2017–2019)**	***p*–value**
	***n* = 28**	***n* = 36**	
Age at EA surgery (d)	3 (0–58)	3.5 (1–54)	0.17
NICU time (d)	9 (3–126)	8 (3–81)	0.91
LOS (d)	20 (10–159)	25.5 (10–178)	0.14
Leakage	3 (11%)	6 (17%)	0.72

*EA, esophageal atresia; LOS, length of hospital stay*.

**Table 3B:** Surgical data of group 2 (2017–2019).

**Variable** **(median, range)**	**No-PPT,** ***n* = 22**	**PPT,** ***n* = 14**	***p*-value**
Age at EA surgery (d)	3.5 (1–54)	3.5 (1–35)	0.28
NICU time (d)	8 (3–81)	11 (3–59)	0.83
LOS (d)	24 (13–178)	28 (12–93)	0.81
Leakage	3 (14%)	3 (21%)	0.66

*PPT, primary posterior tracheopexy; EA, esophageal atresia; LOS, length of hospital stay*.

**Table 4:** Tracheomalacia evaluated during bronchoscopy before EA repair in group 2.

**Variable**	**Group 2** **(2017–2019)**
	***n* = 36**
No TM	5 (14%)
TM mild	15 (42%)
TM moderate/severe	13 (36%)
No TM evaluation possible	3 (8%)

*EA, esophageal atresia; TM, tracheomalacia*.

Patients that underwent PPT were operated at a median age of 3.5 days (range 1–35 days). Surgery was postponed due to respiratory instability in one patient of group 2, a premature neonate of 1,050 g (28.7 weeks). One patient of group 2 was operated in an emergency setting because of a gastric perforation and pneumothorax that occurred during CPR shortly after birth.

### Overall Respiratory Outcome

In group 1, 11 patients (39%) experienced at least one BRUE, compared to seven patients (19%) in group 2 (*p* = 0.09). RTIs requiring antibiotics within the first year of life occurred significantly less often after introduction of PPT (group 1 vs. group 2; 61 vs. 25%, *p* = 0.004; Table 5).

**Table 5:** Respiratory outcome in group 1 and group 2.

**Variable**	**Group 1** **(2014–2016)**	**Group 2** **(2017–2019)**	***p*-value**
	***n* = 28**	***n* = 36**	
BRUE	11 (39%)	7 (19%)	0.09
RTI <1 year	17 (61%)	9 (25%)	0.004^*^

*BRUE, brief resolved unexplained event; RTI, respiratory tract infection requiring antibiotics*.

* *Indicating statistical significance*.

In group 1, three patients underwent an aortopexy at the median age of 12 days (range 12–29). In two of these three patients, severe TM was evaluated preoperatively with bronchoscopy. In one patient after aortopexy, TM persisted and consequently a posterior tracheopexy was performed.

In group 2, redo tracheopexy was not warranted in any of the PPT-patients. Bronchoscopy was incomplete in three patients of group 2 and TM could not be evaluated, because spontaneous breathing during bronchoscopy was impaired due to ventilation problems. Of these three patients, one had to undergo a secondary posterior tracheopexy and another patient was treated by aortopexy.

Four patients, two in each group, all with multiple comorbidities, needed a tracheostomy. One patient with a tracheostomy and Down's syndrome in group 1, died during follow-up due to accidental decannulation.

### Respiratory Outcome After PPT Introduction

Subgroup analysis of group 2 showed occurrence of BRUEs in one patient (7%) in the PPT-patients group vs. 6 patients (27%) in the no-PPT patients group (Table 6). This difference, however, was non-significant. The one patient with BRUEs after PPT had two tracheoesophageal fistulas (the distal fistula was located in the carina, and the proximal fistula in the middle part of the trachea) and a severe TM in the middle part of the trachea on preoperative bronchoscopy. Therefore, this patient underwent selective PPT only at the level of this middle part of the trachea. Postoperative bronchoscopy in this patient with multiple comorbidities, including a subglottic stenosis and retrognathia, revealed a severe TM in the distal part of the trachea.

**Table 6:** Respiratory outcome in group 2 (2017–2019).

**Variable**	**No-PPT,** ***n* = 22**	**PPT,** ***n* = 14**	***p*-value**
BRUE	6 (27%)	1 (7%)	0.21
RTI <1 year	6 (27%)	3 (21%)	1.0

*PPT, primary posterior tracheopexy; BRUE, brief resolved unexplained event; RTI, respiratory tract infection requiring antibiotics*.

RTIs requiring antibiotics within the first year were seen in 21% in the PPT-patients vs. 27% in the no-PPT patients group. One patient in the PPT-patient group experienced postoperative respiratory distress caused by a suture granuloma. After removal of the granuloma by bronchoscopy, no further respiratory problems occurred.

## Discussion

This is the first prospective study to evaluate respiratory outcome after thoracoscopic primary posterior tracheopexy in EA patients with tracheomalacia.

This novel PPT technique decreases the number of respiratory tract infections (RTIs) in EA patients with moderate or severe TM. The number of BRUEs also seemed to decrease after introduction of PPT, although this was not statistically significant. Furthermore, the PPT-procedure takes only short additional operative time and there were no differences in hospital length of stay, NICU stay and postoperative leakage between the PPT-group and the no-PPT group.

Respiratory morbidity in EA is very common during early childhood (13). EA patients often suffer from RTIs during the first year of life (14–17). This is in line with our findings, showing 61% of patients with RTIs within the first year of life before introduction of PPT. After the introduction of PPT, RTIs requiring antibiotics were significantly decreased in both EA patients that underwent a PPT, as well as the entire EA cohort (EA patients with and without PPT between 2017 and 2019). Therefore, selecting EA patients with moderate to severe TM for PPT improves the respiratory outcome of EA patients as a whole. In a previous study on PPT (18), a decrease in RTIs was not seen. However, results are difficult to compare, since this study compared preoperative data to postoperative data within a group of 18 patients and follow-up duration was shorter (5 months).

Another possibly life-threatening aspect of respiratory morbidity is posed by BRUEs. Although the decrease in number of BRUEs after the introduction of PPT from 39 to 19% seemed evident, it was not statistically significant. However, this may be explained by the small number of patients. In the study by Shieh et al. (18), a decrease in BRUEs was shown (*p* = 0.049). However, in this study, almost 30% of patients were re-operated for persistent collapse of the trachea.

In a previous study (10), we showed thoracoscopic PPT to be feasible and safe, with favorable short-term outcome. During the longer follow-up in this study, one patient experienced respiratory problems, caused by a suture granuloma. After bronchoscopic removal of the granuloma, no more respiratory problems had occurred. Flexible bronchoscopic visualization during posterior tracheopexy may prevent this type of complication from occurring. Therefore, this has now been implemented as routine procedure during PPT in our center. Moreover, this may also optimize positioning of the sutures in the tracheal wall.

In three patients, TM could not be evaluated due to ventilation difficulties during bronchoscopy. In these patients, PPT was not performed since the extent of TM was unknown. In this study, in two out of three patients, a secondary surgical intervention for severe TM was warranted. In these patients posterior tracheopexy was challenging because of multiple adhesions and risk of damaging the esophageal anastomosis. Therefore, median duration of secondary tracheopexy takes significantly longer than PPT (hours vs. minutes) and can be associated with complications (3, 10).

Attention for respiratory morbidity in EA patients, and especially for TM, has raised over the past few years (1, 19, 20). Therefore, routine preoperative rigid bronchoscopy is performed in all EA patients in the University Medical Center Utrecht, Wilhelmina Children's Hospital since 2015. The introduction of a standardized scoring system for TM in 2017 (11) by the dedicated congenital esophageal and airway team has led to increased awareness and improved recordings at our center.

A limitation of this study was that the rigid bronchoscopy was not repeated after PPT. In order to evaluate the effect of PPT, collapse of the trachea should be compared by rigid bronchoscopy before and after PPT. This would, however, require a second anesthesia for rigid bronchoscopy since spontaneous breathing is not possible directly after thoracoscopic EA repair and PPT. Therefore, our congenital esophageal and airway team has chosen not to evaluate the trachea by means of a second invasive procedure.

Although the data of the no-PPT patients was assessed retrospectively, the variables of interest were collected prospectively at standardized moments for all patients. Standardized questionnaires were not used, however, structured interviews regarding gastrointestinal and respiratory symptoms were conducted in every patient at our Congenital Esophageal and Airway outpatient clinic.

Another limitation is that no standardized scoring system was used during preoperative bronchoscopy in group 1. However, the baseline characteristics were similar within the two groups and therefore we expect that there are no significant differences on severity of tracheomalacia between the two groups.

Preferably, a prospective trial in EA patients with moderate or severe TM randomizing for PPT or no-PPT is needed to provide the best level of evidence. This comparative study shows the potential benefits of primary posterior tracheopexy in EA patients with concurrent moderate to severe tracheomalacia with a low complication rate.

## Conclusions

In conclusion, this study shows that thoracoscopic primary posterior tracheopexy during esophageal atresia repair can significantly decrease respiratory tract infections that require antibiotics during the first year of life. The clinical impact of reducing respiratory tract infections combined with the relatively minimal additional operating time and safety of PPT may outweigh the risk of overtreatment. This, however, should be evaluated in an international, multicenter randomized controlled trial comparing PPT to no-PPT in neonates with EA.

Naturally, this advanced technique should only be performed in centers with a team of experienced pediatric upper GI- and airway surgeons, otolaryngologists, pulmonologists and anesthesiologists.

## Data Availability Statement

Requests to access these datasets should be directed to m.y.a.lindeboom@umcutrecht.nl.

## Author Contributions

ESTS conceptualized and designed the study, collected the data, carried out the initial analysis, drafted the initial manuscript, and reviewed and revised the manuscript. ML conceptualized and designed the study, contributed to the writing, supervised the data collection and the progress, and reviewed and revised the manuscript. ST and DZ provided input to the study, supervised the data collection and the progress, and reviewed and revised the manuscript. JV, AB, SC, and HA reviewed the study design, contributed to the interpretation of data, and reviewed and revised the manuscript. All authors approved the final manuscript as submitted and agree to be accountable for all aspects of the work in ensuring that questions related to the accuracy or integrity of any part of the work are appropriately investigated and resolved.

## Conflict of Interest

The authors declare that the research was conducted in the absence of any commercial or financial relationships that could be construed as a potential conflict of interest.

## Publisher's Note

All claims expressed in this article are solely those of the authors and do not necessarily represent those of their affiliated organizations, or those of the publisher, the editors and the reviewers. Any product that may be evaluated in this article, or claim that may be made by its manufacturer, is not guaranteed or endorsed by the publisher.

## Abbreviations

EA: esophageal atresia
TM: tracheomalacia
PPT: primary posterior tracheopexy
BRUE: brief resolved unexplained event
RTI: respiratory tract infection.
